# Continuous Rural High School Educational Outreach and Lasting Impact on Health Care Career Attitudes: Qualitative Pilot Study

**DOI:** 10.2196/70505

**Published:** 2025-07-14

**Authors:** Rebecca Bolen, Meagan Flesch, Jerrica Dennis, Logan Shouse, Mark Payton, David Ross

**Affiliations:** 1 Department of Tracks and Special Programs, College of Osteopathic Medicine, Rocky Vista University, 8401 S Chambers Rd, Englewood, CO, 80112, United States, 13033732008; 2 Department of Biomedical Science, College of Osteopathic Medicine, Rocky Vista University, Parker, CO, United States; 3 Department of Tracks and Special Programs, College of Osteopathic Medicine, Rocky Vista University, Parker, CO, United States

**Keywords:** rural health, health disparities, career pathways, high school students, education, healthcare workforce

## Abstract

**Background:**

Rural communities face persistent challenges in recruiting and retaining health care professionals. Research has shown that individuals from rural backgrounds are more likely to return to practice in these areas, yet most existing pipeline programs focus on undergraduate and medical students rather than high school students. Early exposure to health care careers is essential, as many students have already selected their career paths by the time they enter college. A previous study conducted in 2020 analyzed the effects of a single educational workshop at a rural high school in New Hampshire. The results suggested that students had a better understanding of the health care field.

**Objective:**

This study evaluates the impact of an ongoing educational outreach program designed to introduce rural high school students to diverse health care professions.

**Methods:**

This study was conducted at West Grand High School, a rural high school in Kremmling, Colorado, between September and December 2023. The intervention consisted of 4 monthly sessions, each focusing on a different medical specialty—primary care, sports medicine, dermatology, and neurology. These sessions, led by second-year medical students, provided an overview of common conditions, treatment approaches, and various health care roles involved in patient care. Participants completed pre- and postsession surveys assessing their interest in health care careers, perceived barriers, and likelihood of returning to their rural hometown to practice. A follow-up survey was conducted 4 months after the final session to assess long-term impact.

**Results:**

While individual session surveys showed no significant changes, overall interest in and likelihood of pursuing a health care career increased significantly over the course of the presentation series (*P*=.03 and *P*=.04, respectively). However, there was no significant change in students’ likelihood of returning to their rural hometowns to practice or their perceived access to resources for a health care career. Financial constraints (43/66, 65%) were identified as the most significant barrier, followed by lack of exposure (19/66, 29%) and support (17/66, 26%), while interest and education were least likely perceived as obstacles.

**Conclusions:**

This study highlights the importance of early and sustained outreach efforts in rural communities to increase awareness of diverse health care career pathways. While short-term educational interventions can positively influence career interest, long-term mentorship and structured support systems are essential for fostering a sustained commitment to rural health care careers. Future initiatives should integrate financial counseling, ongoing mentorship, and collaborations with existing rural pipeline programs to enhance the effectiveness of such interventions.

## Introduction

Rural and remote populations frequently contend with a lack of health care providers, reduced access to specialty services, and health outcomes that lag behind those in urban centers [1]. A Merritt Hawkins White Paper in 2018 reported that 20% of the US population lives in rural areas, yet only 9% of US physicians practice in these communities [2]. Limited health care infrastructure, long travel distances for care, and professional isolation all contribute to the difficulty in recruiting and retaining qualified health care professionals in rural areas [2].

Research consistently shows that health care professionals who have lived in or originated from rural areas are significantly more likely to return to these communities to practice [3-5]. As a result, early exposure to health care careers for rural students is a critical strategy to bolster the rural health workforce. Without targeted interventions, students in rural communities may lack awareness of health care career pathways or perceive such careers as unattainable, perpetuating the cycle of inadequate health care services and an insufficient number of health care providers. Structural and socioeconomic barriers, such as fewer academic role models and geographic isolation, can further limit students’ access to the information and mentorship necessary to pursue health-related careers [6]. However, by implementing continuous educational outreach initiatives, we can bridge the gap between rural high school students and health care–related professions such as physicians, nurses, various technicians, and related health care field careers.

Early engagement is especially important because many students make key career decisions during high school [7,8]. Research indicates that structured exposure to health care professions before college can significantly enhance students’ interest, motivation, and self-efficacy in pursuing those careers [8,9]. These programs not only provide critical information about health professions and educational pathways but also offer students the opportunity to connect with real-world role models, demystify professional stereotypes, and envision themselves as future health care providers. For example, outreach programs that include hands-on experiences, simulations, facility tours, and personal stories from health professionals have been shown to increase students’ interest in health careers, particularly in rural and underserved settings [10-14].

Many existing “pipeline” programs support undergraduate and medical students in pursuing careers in rural health care, but there is a deficit in programs that target rural high school students [15,16]. These students are at a formative stage where exposure can have a lasting impact on their academic and career trajectories. Without outreach during this period, we risk missing students who may never pursue postsecondary education due to a lack of early encouragement or awareness of viable career options [7,17].

The benefits of inspiring rural high school students to pursue careers in health care are twofold. First, it addresses the shortage of health care professionals in rural areas. By encouraging students from these communities to pursue health care careers, we increase the likelihood that they will return to their hometowns to practice, improving access to health care for the local population [18]. Furthermore, the early introduction of students to a diverse range of career paths within health care, beyond traditional roles such as doctors and nurses, can help students align their interests, strengths, and passions with a particular career path, allowing them to make informed decisions about their future careers [17].

Previous studies have demonstrated the effectiveness of educational outreach initiatives. For example, a 2020 project in New Hampshire provided rural high school students with a day of health care workshops and mentorship opportunities, fostering connections between students and health care professionals [19]. Another such initiative is the Canadian Healthcare Travelling Roadshow, a short-term yet highly interactive program that visits rural communities and delivers hands-on sessions designed to inspire youth toward health careers. Following participation, students reported increased interest in health professions and a stronger sense of connection with health care providers [13]. Similarly, a growing body of literature supports the value of high school–level outreach in increasing health career consideration, particularly in underserved populations [7-10]. However, many existing programs lack continuity and structured follow-up, limiting their long-term impact. Outreach programs that are sustained over time, rather than limited to a single event, may have a greater impact by building mentorship relationships, reinforcing career aspirations, and providing ongoing academic support.

This study aims to analyze how more continuous educational outreach initiatives affect rural high school students’ attitudes toward a career in health care. Through multiple sessions over a longer period than previously outlined initiatives, it is anticipated that ongoing mentorship and education will inspire a lasting interest in health care professions. By empowering students and equipping them with knowledge, skills, and exposure, we can cultivate a new generation of health care professionals who are motivated to serve their communities.

## Methods

### Recruitment

This study was conducted from September to December 2023 at West Grand High School (WGHS) in Kremmling, Colorado. WGHS was selected due to its rural location. Kremmling, a town of less than 1500 residents in the Rocky Mountains, is at least a 2-hour drive from any larger populated area and 3 hours from a metropolitan district. WGHS ranks 245 of 334 Colorado high schools and 13,242 of 17,655 nationally [20]. Minority enrollment is 36%, and 44% of students are economically disadvantaged [20]. The school’s total enrollment for grades 9‐12 is 112 students [20]. Given its rural setting, many students may have limited exposure to health care professionals or career pathways in health care–related fields. Understanding this context is essential in evaluating the impact of the intervention.

Participants were recruited based on their interest in the program, with all grade levels invited. Each session included approximately 8‐10 students (~9% of the student body), all of whom were female, ranging from freshmen to seniors. The same students attended each session throughout the study.

### Program Design and Data Collection

The outreach program was structured as a health specialty educational series designed to introduce students to a variety of health care careers and training pathways. Data collection occurred over 4 sessions, each spaced approximately 1 month apart. Each session included a 20-minute presentation focused on a specific specialty: primary care, sports medicine, dermatology, or neurology. These topics were selected not only based on presenter interest but also for their ability to incorporate a diverse range of health care careers, ensuring that students could explore multiple professional roles beyond physician pathways. In addition, faculty insights were gathered to align the topics with students’ expressed interests. The sessions were specifically designed for a high school level of education, using accessible language, interactive activities, and relatable role models to foster engagement. The program built upon existing college counseling efforts at the school, integrating health care–specific content to complement and expand the resources already available to students.

Presentations were delivered by 4 second-year medical students from Rocky Vista University and were designed to highlight common conditions within each specialty while showcasing the interdisciplinary nature of health care. Each session emphasized the roles of various professionals involved in patient care, including physicians (MD or DO), physician assistants, nurse practitioners, nurses, athletic trainers, aestheticians, psychologists, technicians, and other allied health professionals. Discussions covered the educational and training requirements for each profession to provide students with a broader understanding of health care career pathways.

To enhance engagement, each session incorporated real-world clinical case presentations to demonstrate how different health care professionals collaborate in patient care. These case-based discussions encouraged critical thinking and allowed students to visualize the diverse roles within the health care system.

Before and after each presentation, participants completed anonymous voluntary surveys assessing their interest in health care. All surveys consisted of the following five questions: (Q1) How likely are you to stay in or return to your hometown to pursue your career?; (Q2) How interested are you in a career in health care? (Q3) What is the likelihood that you will go into a career in health care?; (Q4) What barriers affect the likelihood that you will go into a career in health care?; and (Q5) Do you feel that you have the resources (support, money, knowledge, etc) to pursue a career in health care?

Responses to questions 1, 2, 3, and 5 were based on a Likert scale from 1 to 5, corresponding to the answer choices of strongly disagree, disagree, neither agree nor disagree, agree, and strongly agree. Q4 allowed for participants to select any of the following that apply: money, educational background, lack of social support or family support, knowledge or exposure to health care, interest in health care.

In addition, the postpresentation surveys included five open-ended questions: (1) What did you like about this workshop?; (2) What did you dislike about this workshop?; (3) What questions or comments do you still have?; (4) Does anyone in your family or close friends work in the health care field? If so, who and what do they do?; and (5) Do you have any experience working or volunteering in the health care field? If so, what is your experience?

### Statistical Analysis

A 2-factor ANOVA was run for each of the 4 Likert scale questions with month, presurvey, and postsurvey as the factors of interest. Pre-post analyses were performed for each month and longitudinally for the entire presentation series. *P* values, means, SEs, and sample sizes were calculated for each comparison. If a comparison between months given pre-post data was significant (*P*<.05), pairwise comparisons were made.

For Q4, contingency tables were constructed to examine potential relationships between students’ likelihood of entering the health care field and each listed barrier. Contingency tables allow for the evaluation of interrelationships between categorical variables. A *χ*^2^ test was then applied to determine statistical significance.

### Ethical Considerations

This survey study was approved by the Rocky Vista University Institutional Review Board for participation by high school–aged students (approval #2023-150). Assent forms, which outlined the purpose and voluntary nature of the study, were completed by students, and parental consent was obtained prior to the presentation series. The original informed consent permitted secondary data analysis without the need for additional consent. All survey responses were collected anonymously, with no identifying information recorded. Students were informed of their right to withdraw from the study at any time without forfeiting the opportunity to attend the presentations. No monetary or material compensation was provided for participation.

## Results

**Figure 1.**
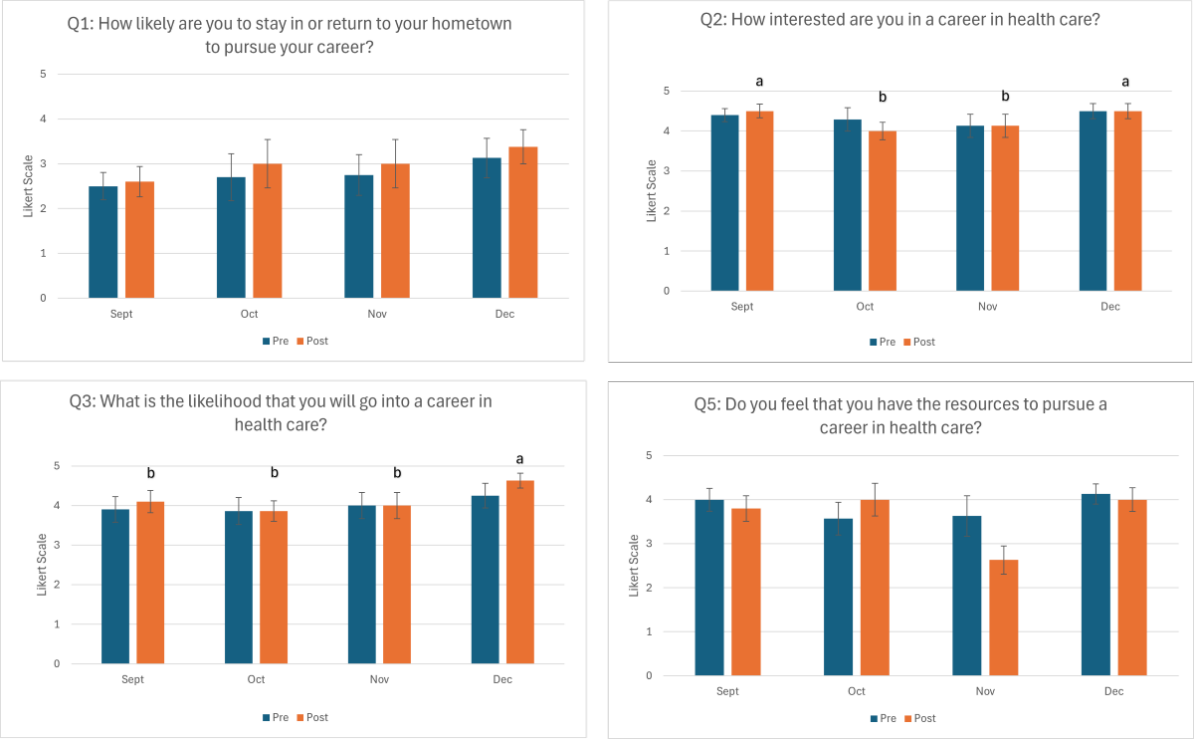
Representation of student responses over the 4 months of presentations: September (n=10), October (n=7), November (n=8), December (n=8). A self-reported Likert scale was used, with 5 being “strongly agree” and 1 being “strongly disagree.”

Analysis of pre- and postpresentation survey responses showed no significant changes within each individual session. However, responses to Q2 and Q3 (interest in a career in health care and the likelihood of going into a career in health care) indicated a significant increase over the course of the presentation series (*P*=.03 and *P*=.04, respectively), as seen in Figure 1. There was no significant change in the likelihood of the students staying or returning to their hometown to pursue a career (Q1) or in their feelings about having the resources to pursue a career in health care (Q5; Figure 1).

Analysis of the barriers to pursuing a health care career (Q4) in relation to Q3 revealed significant findings. In order of likelihood deemed as a barrier was money > exposure > support > interest, with education and interest being significantly less likely to be considered barriers compared to the first three (Table 1). However, statistical significance was only present as a negative correlation regarding interest and education, suggesting that students did not perceive these as barriers to pursuing a health care career.

**Table 1.** Representation of student-reported barriers to pursuing a career in health care over the whole 4-month period. In order of most perceived as a barrier to least: money > exposure > support > education > interest.

Q4: What barriers affect the likelihood that you will go into a career in health care?	Responses, (n/N, %)
Money	43/66, 65%
Educational background	10/66, 15%
Exposure	19/66, 29%
Lack of support	17/66, 26%
Interest	3/66, 5%

Several key themes emerged from student responses to the open-ended questions. Students expressed enthusiasm for learning about health care careers beyond becoming a physician. Many were unaware of the wide range of available roles, highlighting the need to further expand the series to explore additional career paths. Students also provided feedback on areas for improvement. Several mentioned a desire for more interaction with the lecture series team, as the separate presentation format limited engagement with all presenters. In addition, students requested a more detailed exploration of each health care career, which may necessitate extending the series if high school schedules allow. About half of the students reported having a family member or acquaintance in health care, raising questions about how to better engage those without prior exposure. Most students also reported little to no health care–related work or volunteer experience, with many citing personal patient experiences as their primary exposure to the field.

## Discussion

### Principal Findings

Previous studies have primarily focused on short-term outreach programs that rely on isolated lectures or single-day simulations [13,19]. While these models showed to spark initial interest, their limited duration may lack the continuity needed to sustain long-term impact. In contrast, this program emphasized consistent exposure and ongoing engagement through a series of sessions and hands-on experiences. This repeated contact not only reinforces student interest but also fosters mentorship and deeper career exploration, potentially leading to more lasting shifts in students’ aspirations toward health care professions.

The data demonstrate that the educational outreach program produced a statistically significant increase in interest in and likelihood of pursuing health care careers among rural high school students. Specifically, interest in pursuing health care careers (Q2) significantly increased from October to December, but not from September to December. This lack of significance over this study period may be attributed to fluctuations in sample size between September and December. We did observe a linear increase in interest from October to December, suggesting that consistent exposure to the intervention may contribute to sustained interest. As previously discussed, research shows that early and sustained exposure to health care significantly increases student interest, especially when introduced before college [8]. The consistent engagement from October to December suggests that continued exposure during this stage of education can serve as an effective early intervention, inspiring students to consider careers in health care. This approach may help address the rising demand for physicians in rural areas, as students from these communities are more likely to return and practice in their hometowns [3-5].

The likelihood of pursuing health care careers (Q3) increased over the entirety of the program. These findings support the potential benefit of implementing similar health care educational series in other rural high schools. Furthermore, the results showed no significant difference in responses from the beginning to the end of each individual presentation. This suggests that cumulative exposure to various health care specialties and career pathways, rather than interest in any single specialty, increased overall interest in the health care field. This impact may, in part, be attributed to the progressive development of mentorship and a growing sense of connection fostered throughout the educational series. This evolving rapport may have contributed to making a career in health care feel more accessible and attainable. Openly sharing experiences can help humanize the profession. Future research could explore whether specific specialties or health care roles have a greater impact on student engagement and how continued exposure shapes career aspirations over a more longitudinal time.

It is important to note that an increased interest in health care careers does not necessarily correspond to an increased interest in rural health care careers. While a rural background is a known factor influencing the choice to practice in rural settings, it is not a guarantee [3]. Many rural students pursue higher education with the intent of leaving rural areas. Literature has demonstrated that mentorship and ongoing exposure to rural practice settings are essential to maintaining interest in returning to rural health care [7]. Future iterations of this program could incorporate structured mentorship with local rural health care professionals and opportunities for hands-on exposure to rural health care settings, reinforcing the connection between career interest and rural practice.

The lack of statistically significant change in responses to Q1 (likelihood to stay or return to hometown) and Q5 (perception of having resources to pursue a health care career) is noteworthy. Since students’ likelihood to stay or return to their hometown remained unchanged, this suggests that their career interest was independent of geographic location. Future interventions could address this by incorporating discussions on the unique opportunities and challenges of rural health care practice, including scope of practice, job demand, and lifestyle considerations. Increasing awareness of rural-specific health care career pathways, financial incentives for rural providers, and community impact could potentially enhance students’ interest in remaining in their hometowns and addressing the shortage of rural health care professionals.

Similarly, the lack of change in students’ perceptions of having the necessary resources to pursue a health care career (Q5) suggests that the presentations did not alleviate concerns about financial and educational barriers. Many students expressed concerns about the cost of health care education, particularly medical school. Expanding the curriculum to include sessions on scholarships and loan repayment programs could help demystify the financial aspects of health care training and provide students with concrete strategies to navigate educational barriers.

Regarding Q4 (barriers to pursuing a health care career), analysis revealed a negative correlation between perceived barriers related to interest or education and the likelihood of entering health care. This suggests that students did not view these factors as significant obstacles, which may reflect a growing confidence in their ability to pursue a health care career following the program.

To assess the long-term impact of this intervention, the same questionnaire was administered to students 4 months after the final presentation. The analysis compared responses from the initial survey in September to those from the follow-up questionnaire in April. The results indicated no significant change in students’ responses over this period. This can be interpreted in several ways. One plausible explanation is a Type II error due to small sample size, which limits statistical power and may have resulted in a false negative. Expanding the program to a larger student population across multiple schools could provide a more robust evaluation of its effectiveness. Another possible explanation is the need for sustained exposure and reinforcement. Literature suggests that continued mentorship and health care exposure are necessary to solidify long-term interest in health care careers, particularly in rural settings [7]. Without additional interventions, students may not receive sufficient reinforcement of their interest in health care. To overcome this phenomenon, the program will need to be expanded over a longer duration or provide additional opportunities for students to learn outside of the presentation hours. The Canadian Healthcare Travelling Roadshow demonstrated that hands-on exposure to diverse health care fields can significantly boost student interest in medical careers [13]. Drawing on this model, this program added a full-day experience at the medical school of the presenters, offering interactive stations such as ultrasound, intubation, cardiopulmonary resuscitation (CPR), and phlebotomy. This immersive component allowed students to envision themselves in health care roles through direct engagement. While not formally studied, student feedback indicated that the experience greatly enhanced their enthusiasm for pursuing careers in health care.

The findings regarding the open-ended questions highlight important areas for refinement and expansion in future lecture series. Many students found brief discussions on financing a medical education particularly valuable, as they were previously unaware of loan options, scholarships, and repayment strategies. The strong interest in financial education suggests that a dedicated lecture on funding options could play a crucial role in reducing perceived barriers to a health care career. Similarly, the enthusiasm for diverse health care career paths reinforces the importance of broadening the scope of future presentations to ensure students are aware of all available opportunities. Engaging students with no prior connection to health care remains a challenge, but targeted outreach efforts, such as early advertising or school assembly presentations, may help attract a more diverse audience. In addition, students expressed a desire for greater interaction with presenters, as the separate presentation format limited engagement. Personal storytelling has been shown to enhance student interest in health care careers, particularly within rural and underserved communities [13]. To address this, future series will incorporate an introductory question-and-answer session, allowing presenters to share their diverse journeys and foster a more interactive learning environment.

A key component of the program was providing students with mentorship opportunities. While mentorship was not a formalized aspect of the study, students were given the emails of all medical student presenters and were encouraged to reach out with questions. If a medical student could not answer a question, they connected students with a relevant health care professional who could provide guidance. Follow-up plans are also in place to sustain and expand this program. The initiative has been passed down to the next class of medical students to ensure continuity and further develop the program across additional rural high schools in Colorado. In addition, connecting students to existing mentorship programs through organizations such as the Colorado Area Health Education Center could provide more structured career development opportunities. This could be integrated into the series by incorporating a lecture, as discussed in Q4, that informs students about additional volunteer, shadowing, and health care exposure opportunities. Future research could evaluate the long-term impact of such mentorship connections on students’ health care career trajectories.

Limitations include a small, fluctuating sample size and data collection from only one rural high school. Expanding this program to additional rural high schools would allow for a broader evaluation of its impact across different communities and a more diverse student population. Future research could also explore how different program components (eg, mentorship, financial education, and hands-on exposure) contribute to student engagement and career decision-making.

Although this study includes 4 sessions over 4 months, it represents the first phase of a continuous outreach model intended to be repeated annually and expanded to additional rural high schools. This sustained approach aims to create a lasting impact by reinforcing health care career interest over time through ongoing exposure, mentorship, and community engagement. Overall, the findings suggest that educational outreach programs like this health care specialty series can provide valuable career insight, mentorship, and resources to rural high school students. By refining and expanding such initiatives, we can continue to foster student interest in health care careers, address barriers to entry, and ultimately strengthen the rural health care workforce.

### Conclusion

This study underscores the value of early and sustained outreach in encouraging rural high school students to explore health care careers. By providing repeated exposure, practical skill-building, and relatable role models, the program fostered increased interest in the field. While short-term interventions may spark initial curiosity, long-term engagement, through mentorship, structured support, and clear educational pathways, is key to sustaining student commitment, particularly to rural health care. Although some measures, such as intent to return to rural communities or perceived access to resources, showed limited change, these findings point to clear opportunities for program enhancement. With continued development, this initiative can serve as a scalable model to guide rural students toward health professions and address long-standing workforce gaps in underserved areas.

## Abbreviations

WGHS: West Grand High School
